# Supplementary material for: Homeostatic Model Assessment of Insulin Resistance for Predicting the Recurrence of Hepatocellular Carcinoma after Curative Treatment
Source: Int J Mol Sci. 2019 Jan 30;20(3):605. doi: 10.3390/ijms20030605 (PMC6387449; doi:10.3390/ijms20030605)

Supplementary Figure S1

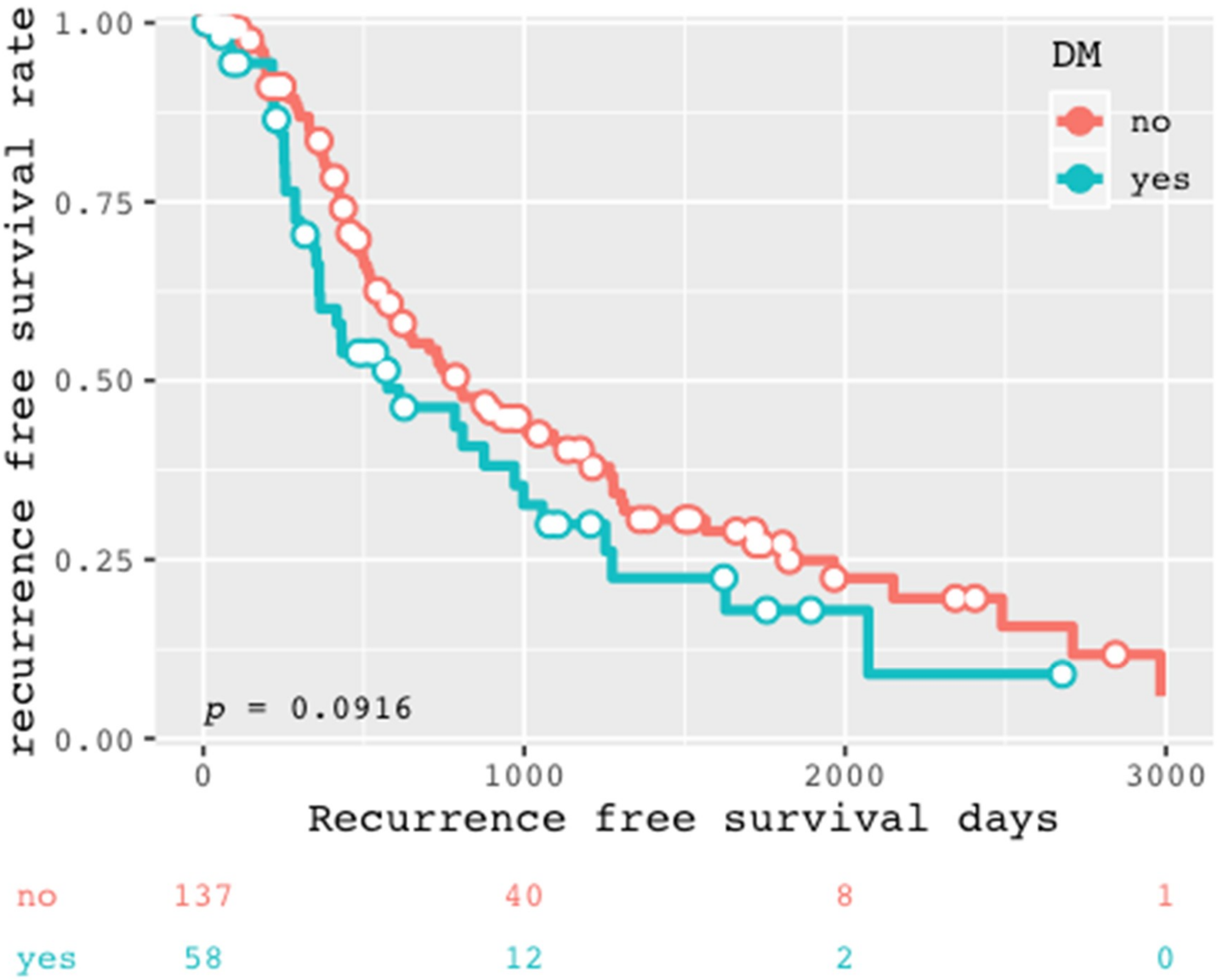

Supplementary Figure S2

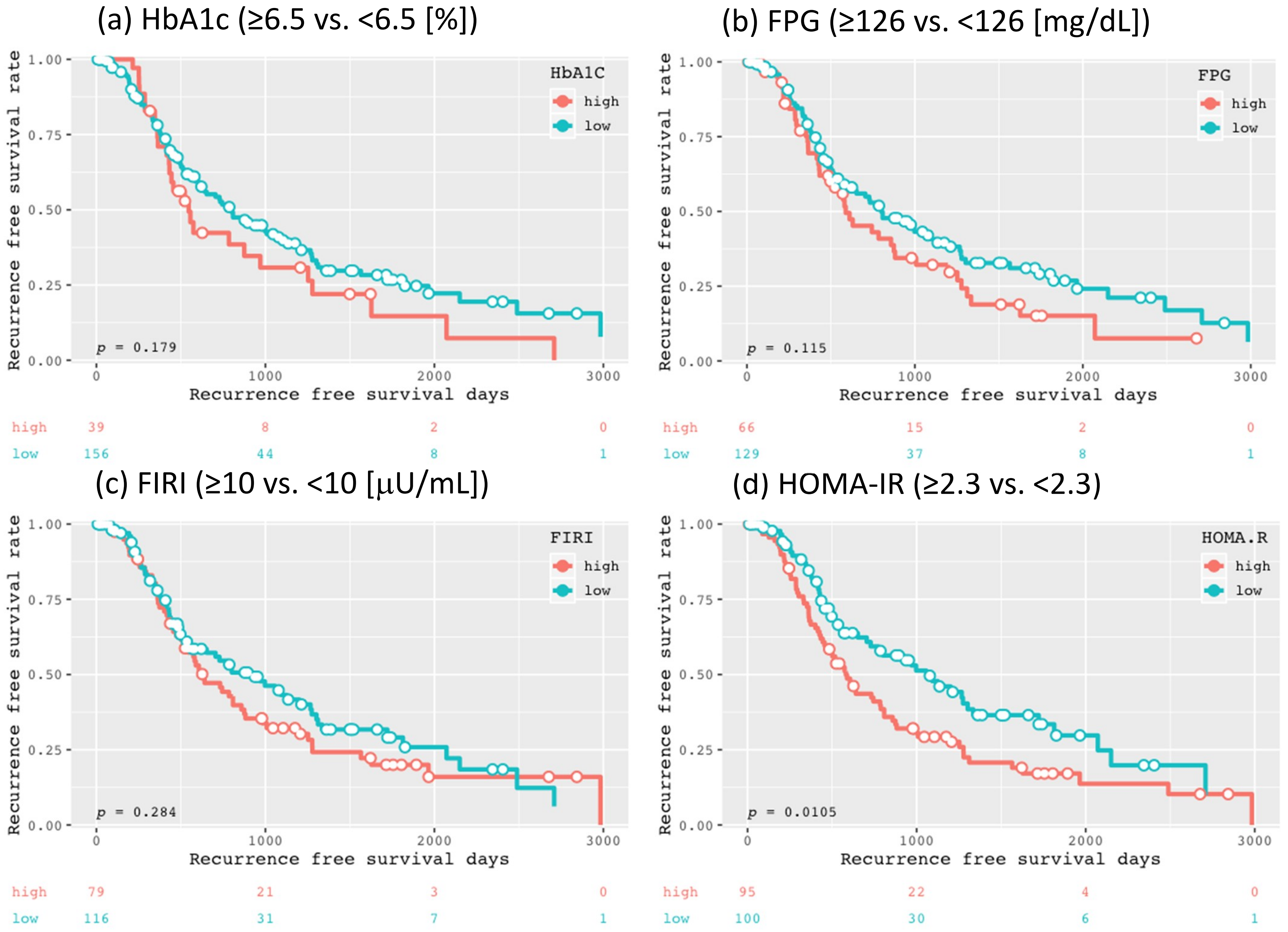

Supplementary Figure S3

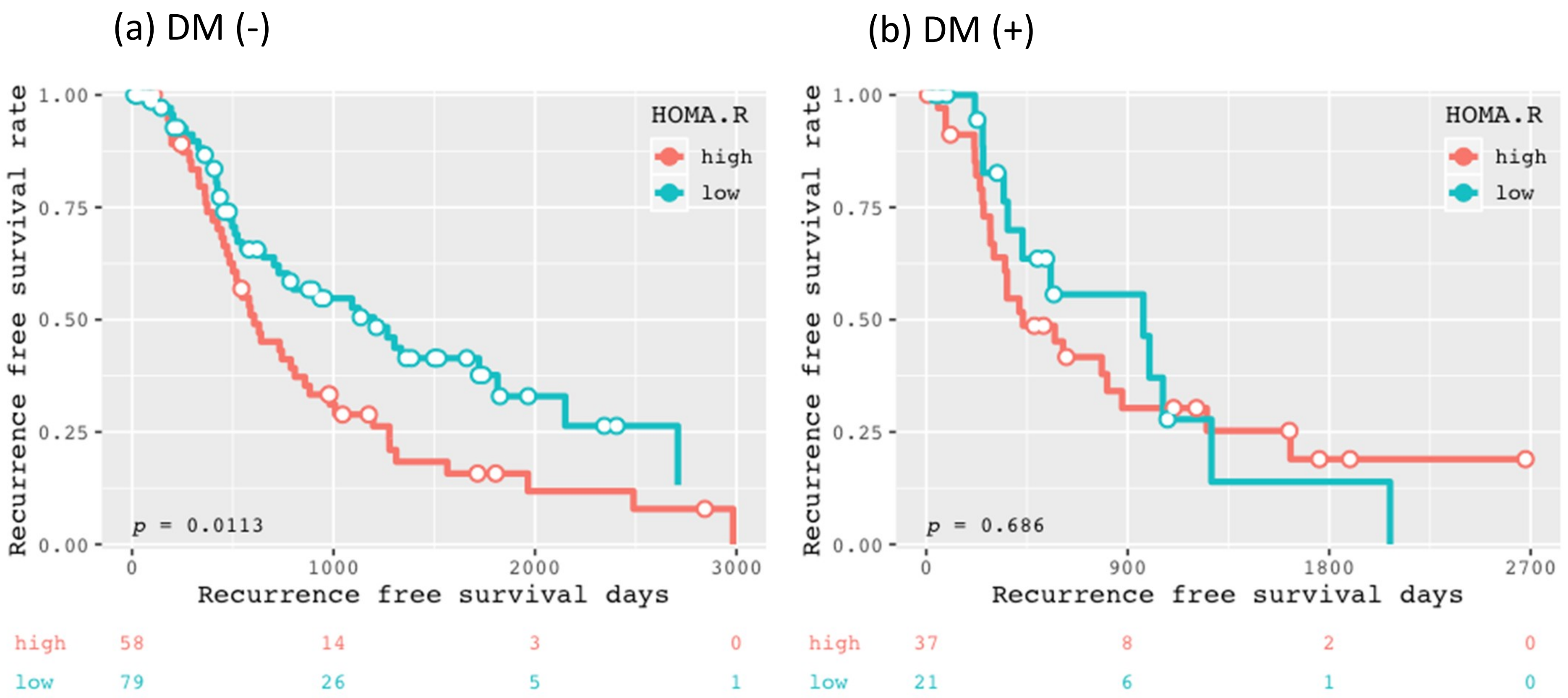

Supplement: Supplementary file 1 [file ijms-20-00605-s001.pdf]
